# Malaria in illegal Chinese immigrants, Italy.

**DOI:** 10.3201/eid0706.010628

**Published:** 2001

**Authors:** A Matteelli, A Volonterio, M Gulletta, L Galimberti, S Maroccolo, G Gaiera, G Giani, M Rossi, N Dorigoni, L Bellina, G Orlando, Z Bisoffi, F Castelli

**Affiliations:** Institute of Infectious and Tropical Diseases, University of Brescia, Italy. forleo@master.cci.unibs.it

## Abstract

A cluster of 22 imported malaria cases, 21 caused by Plasmodium falciparum, was observed among illegal Chinese immigrants in northern Italy in the summer of 2000. The rate of severe disease was high because the patients were not immune and they sought health-care services late in their illness because of their clandestine status. Recognition of the outbreak was delayed because no regional alert system among infectious diseases hospitals was in place.


### Malaria in Illegal Chinese Immigrants, Italy

**Alberto Matteelli,* Alberto Volonterio,† Maurizio Gulletta,* Laura Galimberti,‡ Stefania Maroccolo,' Giovanni Gaiera,¶ Gloria Giani,# Maurizio Rossi,** Nicoletta Dorigoni,†† Luca Bellina,* Giovanna Orlando,# Zeno Bisoffi,§ and Francesco Castelli***

*University of Brescia, Brescia, Italy; †Ospedale Niguarda-Cà Granda, Milano, Italy; ‡University of Milano, Milano, Italy; §Institute for Tropical Medicine, Negrar, Italy; ¶Ospedale San Raffaele, Milano, Italy; #Ospedale Sacco, Milano, Italy; **Ospedale di Menaggio, Menaggio, Italy; and ††Ospedale Santa Chiara, Trento, Italy

A cluster of 22 imported malaria cases, 21 caused by *Plasmodium falciparum*, was observed among illegal Chinese immigrants in northern Italy in the summer of 2000. The rate of severe disease was high because the patients were not immune and they sought health-care services late in their illness because of their clandestine status. Recognition of the outbreak was delayed because no regional alert system among infectious diseases hospitals was in place.

The recent increase of population movements is paralleled by an increase in imported malaria cases in Europe, where malaria is not endemic (1,2). In Italy, migrants and foreign-born people visiting relatives represent an increasing proportion of imported malaria cases (3). Most cases are in persons from malaria-endemic areas. They rarely develop complications because of their semi-immune status and because early diagnosis is usually made on the basis of travel history (4). We describe an unprecedented cluster of malaria cases in immigrants from China. Most of the immigrants originated from the province of Zhe Jiang, which is free from indigenous falciparum malaria (Allan Schapira, pers. commun.); therefore, they had no immunity to the infection.

### Case Reports

During the summer of 2000, several Chinese immigrants were admitted to hospitals in northern and central Italy with a diagnosis of malaria. Twenty-two such cases were observed by the Study Group for International Health of the Lombardy Region clinical network (SIRL) and are described here. Diagnoses of malaria and definitions of species were based on microscopic examination of peripheral blood smears. Twenty cases were due to *Plasmodium falciparum*, one to mixed (*P. falciparum*/*P. ovale*), and one to *P.vivax* infection.

The major epidemiologic and clinical characteristics of the 22 cases are listed (Table). All but one were in immigrants arriving for the first time in Italy. A detailed travel history was obtained from half the patients. Communication was extremely difficult, as none could speak any European language or Arabic, and none was accompanied by family members. Even when translators were used, information was incomplete and incoherent, because of fear of deportation. In most cases the area of origin was the province of Zhe Jiang. These patients reported that at least 3 months had lapsed between their departure from China and arrival in Italy. Traveling by land and sea, these immigrants visited several malarious areas of Southeast Asia and eastern and western Africa. Long stops of several weeks in Laos, Thailand, Myanmar, Bangladesh, Pakistan, Kenya, Tanzania, Ivory Coast, or Cameroon were reported. In at least two cases, Italy was not the country of arrival in Europe: one patient entered France first and another entered Switzerland before being transferred to Italy.

Most patients were young adult men. None reported having taken malaria chemoprophylaxis or adopting other malaria preventive measures. Some had had previous febrile episodes, which had occurred outside Italy and had been treated with unspecified drugs. It was almost invariably impossible to define the lag between onset of current symptoms and the time patients sought care at hospital. The time to diagnosis in hospital was longer than 24 hours in 50% of the cases, and it was longer for the first cases occurring at each hospital.

The clinical picture on admission to hospital was that of a nonspecific febrile illness. The parasite density was >10% in 8 (38.1%) of 21 falciparum malaria cases. In 7 (33.3%) of 21 falciparum cases, the criteria for complicated and severe disease (5) were met. One patient died, and another had neurologic sequelae after recovering from cerebral malaria. Patient 6 was admitted to hospital for headache and fever. His condition deteriorated in 24 hours, with the development of coma and hyperbilirubinemia. He was transferred to an intensive care unit 36 hours after admission. Malaria diagnosis was made 48 hours after admission and specific treatment immediately started. He died on the fourth hospital day from multi-organ failure. The autopsy confirmed massive malaria infection in the brain, lungs, heart, kidneys, spleen, and intestine. Patient 15 had a diagnosis of cerebral malaria (convulsions, impaired consciousness), acute renal

Address for correspondence: Alberto Matteelli, Institute of Infectious and Tropical Diseases, University of Brescia, Piazza Spedali Civili, 1 B 25125 Brescia, Italy; fax: 39-030-303061; e-mail: forleo@master.cci.unibs.it

Table. Epidemiologic and clinical characteristics of 22 Chinese immigrants with malaria in Italy

| No. | Sex, age (yrs) | Origin | Countries visited | Day/ mo of initial visit | Malaria species | Parasite density | Days until hospital dx[a] | Clinical signs & symptoms | Treatment | Outcome |
|---|---|---|---|---|---|---|---|---|---|---|
| 1 | M, 24 | Na | Pakistan | 1/7 | Pf | <1% | 0 | Uncomplicated | Quinine + Doxycycline | Resolved |
| 2 | F, 20 | Na | Na | 3/7 | Pf | 15% | 0 | Uncomplicated | Mefloquine | Resolved |
| 3 | M, 26 | Na | Na | 3/7 | Pf | 15% | 0 | Uncomplicated | Mefloquine | Resolved |
| 4 | M, 20 | Zhe Jiang | Laos, Myanmar, Bangladesh, Pakistan, East Africa | 5/7 | Pf | <1% | 2 | Uncomplicated | Quinine + Doxycycline | Resolved |
| 5 | M, 29 | Southern China | Pakistan, Myanmar, Thailand | 5/7 | Pf | <1% | 1 | Uncomplicated | Quinine + Doxycycline | Resolved |
| 6 | M, 23 | Na | Vietnam, Cambodia, Kenya | 17/7 | Pf | 20% | 2 | Severe | Quinine + Doxycycline | Death |
| 7 | M, 19 | Zhe Jiang | Pakistan, Ivory Coast | 19/7 | Pf | 3% | 2 | Severe | Quinine + antifolics; transfusion | Resolved |
| 8 | M, 33 | Na | Pakistan | 20/7 | Pv | Nd[2] | 0 | Nd[3] | Chloroquine | Resolved |
| 9 | M, 23 | Na | Na | 20/7 | Pf | 2.5% | 0 | Uncomplicated | Mefloquine | Resolved |
| 10 | F, ? | Na | Pakistan | 21/7 | Pf | 20% | 3 | Severe | Quinine + Doxycycline Exchange transfusion | Resolved |
| 11 | M, 26 | Na | Na | 22/7 | Pf | <1% | 0 | Uncomplicated | Quinine | Self discharged |
| 12 | M, 28 | Na | Tanzania | 22/7 | Pf | 11% | 4 | Severe | Quinine + Clindamicin | Resolved |
| 13 | F, 28 | Na | Na | 26/7 | Pf + Po | 10% | 3 | Severe | Quinine + Doxycycline Primaquine | Resolved |
| 14 | M, 24 | Zhe Jiang | Laos, Myanmar, Bangladesh, Pakistan, East Africa | 27/7 | Pf | <1% | 0 | Uncomplicated | Quinine + Doxycycline | Resolved |
| 15 | M, 25 | Zhe Jiang | Laos, Myanmar, Bangladesh, Pakistan, East Africa | 28/7 | Pf | 15% | 8 | Severe | Quinine + Doxycycline Exchange transfusion | Neurologic sequelae |
| 16 | M, 22 | Zhe Jiang | East Africa | 28/7 | Pf | <1% | 0 | Uncomplicated | Quinine | Resolved |
| 17 | M, 18 | Na | Myanmar, Bangladesh, Pakistan, Ivory Coast, France | 2/8 | Pf | <1% | 1 | Uncomplicated | Quinine + Doxycycline | Resolved |
| 18 | F, 19 | Southern China | Kenya | 6/8 | Pf | 1% | 11 | Uncomplicated | Quinine + Doxycycline | Resolved |

[a]A value of 0 indicates diagnosis on the day of admission.

F = female; M = male; dx = diagnosis; mo = month; Na = not assessed; Nd = not determined in a case of *Plasmodium vivax* infection; Pv = *P. vivax*; Pf = *P. falciparum*; Po = *P. ovale*.

Table, continued. Epidemiologic and clinical characteristics of 22 Chinese immigrants with malaria in Italy

| No. | Sex, age (yrs) | Origin | Countries visited | Day/ mo of initial visit | Malaria species | Parasite density | Days until hospital dx[a] | Clinical signs & symptoms | Treatment | Outcome |
|---|---|---|---|---|---|---|---|---|---|---|
| 19 | M, 29 | Na | Pakistan | 7/8 | Pf | 1% | 0 | Uncomplicated | Quinine + Doxycycline | Resolved |
| 20 | F, 24 | Zhe Jiang | Ivory Coast | 6/9 | Pf | 17% | 2 | Severe | Quinine + Doxycycline Exchange transfusion | Resolved |
| 21 | M, 29 | Southern China | Pakistan, Ivory Coast | 11/9 | Pf | <1% | 0 | Uncomplicated | Quinine + Doxycycline | Resolved |
| 22 | M, 18 | Na | Cameroon | 15/11 | Pf | <1% | 0 | Uncomplicated | Mefloquine | Resolved |

[a]A value of 0 indicates diagnosis on the day of admission.

F = female; M = male; dx = diagnosis; mo = month; Na = not assessed; Nd = not determined in a case of *Plasmodium vivax* infection; Pv = *P. vivax*; Pf = *P. falciparum*; Po = *P. ovale*.

failure, and adult respiratory distress syndrom. He received intravenous quinine, doxycycline, and exchange transfusion, and was discharged in apparently good health 24 days after admission. He was readmitted 1 week later with a neurologic syndrome that required a 7-day hospital stay. The final diagnosis was acute psychosis, which was considered a consequence of the previous episode of cerebral malaria. Patients 10 and 20 had cerebral malaria. They were both treated with intravenous quinine, doxycycline, and exchange transfusion and recovered. Patient 12 also had cerebral malaria, was treated with intravenous quinine and clindamicin, and recovered. Patient 7 had acute anemia and received a blood transfusion in addition to quinine and doxycycline. Uncomplicated cases received either quinine or mefloquine, and all recovered. Patient 11 left the hospital on the same day of admission, without completing his malaria treatment course.

### Conclusions

Malaria is a reemerging problem in Italy, with more then 5,000 cases reported from 1986 to 1996 (6). Most cases are imported and present as sporadic disease. The only major cluster of malaria, reported by Orlando and colleagues, was caused by the practice of sharing injection equipment among drug addicts (7). We describe an unprecedented cluster of imported malaria in Chinese clandestine immigrants. Although Italy is a natural point of arrival for immigrants to Europe, it is unlikely that the cluster was limited to Italy: at least two of our patients visited other European countries before entering Italy.

This cluster deserves some discussion. First, malaria prevention in travelers requires active medical interventions and personal motivation for both chemoprophylaxis and prevention of contact with the vector (8). Clandestine migrants are unlikely to receive such medical attention, and none of our patients reported having taken malaria prophylaxis. Second, falciparum malaria in nonimmune persons is potentially fatal and thus needs to be diagnosed and treated early. A diagnostic delay almost certainly occurred among these immigrants, mainly because clandestine immigrants are unlikely to seek prompt medical attention. Although this could not be confirmed, the high parasite density at admission to hospital is an indirect indicator. After hospital admission, diagnostic delay was higher for the first cases and decreased thereafter: in the absence of an alert system each hospital had to learn for itself about the possibility of malaria in Chinese immigrants. Third, travelers from nonmalarious areas pose a difficult diagnostic dilemma to health-care providers, who require a detailed travel history to raise the suspicion of malaria. Language barriers and fear of deportation made this step very difficult in our case list. Even when translators were used, patients were still very reluctant to provide the details of their trip to Italy because of their illegal status. It is also possible that they indeed did not know details about the trip.

The clinical consequence of the factors mentioned above was that at hospital admission cases were more severe than average. The rate of severe malaria in the cluster was 33%, which is much higher than the rates of 1.3% in immigrants and 9.2% in nonimmune Italians recently reported for Lombardy region (9). One patient died, and another one had acute psychosis as a consequence of cerebral malaria.

Italy, from which malaria was eradicated in the 1950s, is susceptible to the reintroduction of the disease because the vector, mainly *Anopheles labranchiae,* is still present at high densities (10). *P. vivax* malaria transmitted by indigenous vectors has recently been documented in Maremma, Italy (11). Italian vectors have been found to be refractory to infection by strains of *P. falciparum* from tropical Africa in in-vitro studies (12). However, the risk of reintroduction of *P. falciparum* strains from Asia, where local vectors are more closely related to the Mediterranean ones, is basically unknown.

Malaria in Chinese immigrants may represent an emerging problem in Europe, one which might increase the rate of severe disease and death from imported falciparum malaria. Interventions to raise awareness of the malaria risk among the Chinese community of clandestine immigrants may not be feasible. To limit the risk for severe disease and death, local health structures in the countries of arrival need to be alert to the possible risk of malaria in febrile illegal immigrants even if they are not coming from classical high-risk areas. This cluster was reported to TropNetEurop, a network which has sentinel clinics throughout Europe, and GeoSentinel, a similar network mainly made up of centers in the United States. A local reporting system could have helped in this specific situation to quickly generate awareness of the outbreak.

### Acknowledgment

We are indebted to Piero Olliaro and Allan Schapira for providing useful information on the epidemiology of malaria in China.

The activities of the Regional Referral Center for Travel Related Diseases are partly supported by grants from the Regione Lombardia.

Dr. Matteelli is responsible for the Centre for International Health at the University Hospital of Brescia. He has field experience as an associate malariologist with the World Health Organization.

### References

1. Legors F, Danis M. Surveillance of malaria in European Union countries. Eurosurveillance 1998;3:45-7.
2. Muentener P, Schlagenhauf P, Steffen R. Imported malaria (1985-95): trends and perspectives. Bull World Health Organ 1999;77:560-6.
3. Sabatinelli G, Romi R, Majori G. Malaria epidemiological situation and assessment of the malaria reintroduction risk in Italy. Giornale Italiano di Malattie Infettive 1998;4:71-87.
4. Castelli F, Matteelli A, Caligaris S, Gulletta M, El-Hamad I, Scolari C, et al. Malaria in migrants. Parassitologia 1999; 41:261-5.
5. World Health Organisation. Severe falciparum malaria. Trans R Soc Trop Med Hyg 2000;94(Suppl 1):S1-S74.
6. Sabatinelli G, Majori G. La sorveglianza epidemiologica della malaria in Italia e aggiornamento della casistica nazionale al 1996. Giornale Italiano di Medicina Tropicale 1996;1:23-7.
7. Orlando G, Marini V, Esposito R, Rancati M, Cargnel A, Almaviba M. An outbreak of *P. falciparum* malaria among drug addicts. Revista Iberica de Parasitologia 1982;1:(Suppl 1):S399.
8. Croft A. Malaria: prevention in travelers. BMJ 2000;321:154-60.
9. Matteelli A, Colombini P, Gulletta M, Castelli F, Carosi G, for the SIRL study group. Epidemiological features and case management practices of imported malaria in northern Italy 1991 - 1995. Trop Med Int Health 1999;4:653-7.
10. Romi R, Pierdominici G, Severini C. Status of malaria vectors in Italy. J Med Entomol 1997;34:263-71.
11. Baldari M, Tamburro A, Sabatinelli G, Romi R, Severini C, Cuccagna G, et al. Malaria in Maremma, Italy. Lancet 1998; 351:1246-7.
12. Ramsdale CD, Coluzzi M. Studies on the infectivity of tropical African strains of *Plasmodium falciparum* to some southern European vectors of malaria. Parassitologia 1975;52:109-11.
